# Genetic Improvement Strategies for Enhancing Feed Efficiency in Aquaculture

**DOI:** 10.3390/biology15201849

**Published:** 2026-10-15

**Authors:** Usman Abdullahi, Ibrahim Kandeh, Jianlin Li, Yuanfeng Xu, Yongkai Tang, Yongfeng Zhao

**Affiliations:** 1 Wuxi Fisheries College, Nanjing Agricultural University, Wuxi 214081, China; aumakanta3@gmail.com (U.A.);; 2 Key Laboratory of Freshwater Fisheries and Germplasm Resources Utilization, Ministry of Agriculture and Rural Affairs, Freshwater Fisheries Research Center, Chinese Academy of Fishery Sciences, Wuxi 214081, China

**Keywords:** feed efficiency, genetic improvement, aquaculture, selective breeding, gene editing

## Abstract

Feed efficiency (FE) is one of the most economically and environmentally important target traits for genetic improvement since feed accounts for 50–70% of total production expenses in aquaculture worldwide. In addition to lowering production costs, reducing feed consumption per unit of biomass output also reduces emissions of nitrogen and phosphorus, which is in line with global sustainability goals for food production. The genetic framework of FE traits, molecular dissection of the targeted loci, conventional selective breeding frameworks, genomic selection, emerging biotechnological applications, major implementation challenges, and prospects are all covered in this review, which summarizes current developments in genetic improvement of FE in aquaculture. We incorporate empirical data from important farmed finfish and crustacean species, emphasizing that FE-related traits show measurable genetic variation available for selection, although reported heritability estimates vary substantially among traits, populations, phenotyping methods, and production conditions. Compared to conventional breeding techniques, the integration of high-throughput phenotyping, genomic prediction and multi-omics approaches has expanded opportunities to characterize FE variation and improve selection accuracy. However, most reported candidate loci and genes remain supported by associative rather than causal evidence, and functional validation in aquaculture remains limited. Integrated breeding approaches therefore offer feasible pathways to mitigate major constraints to genetic improvement of FE, although their practical application differs substantially among species and production systems.

## Simple Summary

Fish farming, or aquaculture, is an important source of protein for people around the world. However, feed is one of the largest costs in fish farming, which can account for up to 70% of total production expenses. This review looks at how scientists are using genetics to improve feed efficiency, the ability of fish to convert feed into body weight, in farmed fish species. Improving feed efficiency reduces production costs and also helps the environment by lowering the amount of nitrogen and phosphorus released into water. The paper examines how feed efficiency is inherited, identifies genes linked to this trait, and reviews breeding methods such as selective breeding, genomic selection, and gene editing that can produce more efficient fish. The findings show that genetic improvement offers a sustainable and cost-effective way to make aquaculture more environmentally friendly while meeting the growing global demand for seafood.

## 1. Introduction

According to a recent report [1], aquaculture and fisheries value chains support over 600 million people globally, while aquaculture accounted for 52.8% of global aquatic-animal production in 2024, up from 51.2% in 2022. Given the comparatively constrained growth of capture fisheries, aquaculture is expected to play an increasingly important role in meeting future global demand for aquatic foods [1]. However, several economic and environmental issues limit aquaculture’s growth, with feed being the single most expensive ingredient [2]. Feed can account for a substantial proportion of production costs in intensive aquaculture systems, although the proportion varies among species, production systems, and geographic regions [2,3]. However, the proportion of feed costs in total production expenses is not uniform across all aquaculture sectors. It varies considerably depending on production intensity, species cultured, and geographic location. For instance, in Nigeria, feed accounts for 60–70% of production costs [4]. In China, feed accounts for over 50% of the total production cost of mandarin fish (*Siniperca chuatsi*) [5]. As output has expanded, the demand for limited fishmeal and fish oil supplies has increased, raising concerns about sustainability [6].

Feed efficiency in aquaculture, which can be defined as the ability of an aquatic animal to convert feed into harvested biomass, is a core trait that directly addresses these challenges [7]. Because feed represents a major component of aquaculture production costs, improvements in FE can provide substantial economic benefits [7]. For example, in Atlantic salmon, improved FE by 2–5% was reported, which would save 8–20 million euros on feed costs in Norway (considering 600,000 tons of feed consumed per year) [8]. Environmentally, improved FE reduces excretion of nitrogen and phosphorus, mitigating eutrophication in aquatic ecosystems, and reduces the requirement for natural resource inputs per unit of production [8,9]. While nutritional and management interventions have delivered FE improvements over the past decades, genetic improvement offers a permanent, cumulative route to sustainability in aquaculture [10]. Recent advances in aquaculture increasingly emphasize the integration of genetic, technological, and management innovations to support more sustainable production systems [11].

Feed efficiency (FE) is an overarching concept describing the efficiency with which consumed feed is converted into biomass and can be expressed using several related metrics. Feed conversion ratio (FCR) is calculated as feed intake divided by weight gain; therefore, a lower FCR indicates better feed efficiency. The feed efficiency ratio (FER), the reciprocal expression of FCR, is calculated as weight gain divided by feed intake, so a higher FER indicates better feed efficiency. Residual feed intake (RFI) is defined as the difference between observed feed intake and the feed intake predicted from specified production and maintenance requirements; lower or negative RFI values indicate that an animal consumes less feed than predicted and is therefore more feed-efficient. The protein efficiency ratio (PER) expresses weight gain relative to protein intake, with higher values indicating more efficient protein utilization [7,12]. RFI is designed to separate observed feed intake from the feed intake expected for specified production and maintenance requirements and has therefore become a useful parameter for genetic investigations in aquaculture. However, its regression-based phenotypic independence from the predictor traits does not guarantee genetic independence; consequently, genetic correlations between RFI and growth or other component traits should be evaluated within each species and population (Table 1) [7].

Available studies report low-to-high heritability estimates for FE-related traits across aquaculture species, indicating exploitable genetic variation while also demonstrating substantial variation among traits, populations, phenotyping methods, and production environments. From fundamental research on genetic architecture to emerging biotechnological applications, this review provides a structured synthesis of current knowledge on the genetic improvement of feed efficiency in aquaculture, while highlighting major challenges and future opportunities for the sector.

## 2. Methods

The present study is a structured narrative review rather than a formal systematic review. A systematic literature search and PRISMA-style flow diagram were used to improve transparency in the identification and selection of the relevant literature; however, no formal risk-of-bias assessment, certainty-of-evidence evaluation, or meta-analysis was undertaken. The relevant literature was searched in Web of Science, Scopus, and Google Scholar because of their broad coverage of aquaculture, genetics, fisheries, and related life-science research.

A pilot search was conducted to assess the suitability of the search terms. Based on this initial search, database-specific search strategies were developed using combinations of terms relating to feed efficiency, genetic improvement, breeding technologies, genomic approaches, and major aquaculture species. The search strategy used for each database was as follows:

Web of Science: TS = (“feed efficiency” OR “feed conversion ratio” OR FCR OR “residual feed intake” OR RFI) AND TS = (“genetic improvement” OR “selective breeding” OR “genomic selection” OR “marker-assisted selection” OR MAS OR QTL OR GWAS OR CRISPR OR “gene editing”) AND TS = (aquaculture OR fish OR crustacean OR salmon OR tilapia OR carp OR shrimp).

Scopus: TITLE-ABS-KEY ((“feed efficiency” OR “feed conversion ratio” OR FCR OR “residual feed intake” OR RFI) AND (“genetic improvement” OR “selective breeding” OR “genomic selection” OR “marker-assisted selection” OR MAS OR QTL OR GWAS OR CRISPR OR “gene editing”) AND (aquaculture OR fish OR crustacean OR salmon OR tilapia OR carp OR shrimp)).

Google Scholar: (“feed efficiency” OR “feed conversion ratio” OR FCR OR “residual feed intake” OR RFI) AND (“genetic improvement” OR “selective breeding” OR “genomic selection” OR “marker-assisted selection” OR MAS OR QTL OR GWAS OR CRISPR OR “gene editing”) AND (aquaculture OR fish OR crustacean OR salmon OR tilapia OR carp OR shrimp).

Because Google Scholar returns a large and dynamically ranked set of results and does not provide the same reproducible indexing structure as Web of Science and Scopus, it was used as a supplementary search source. Results were ranked by relevance, and the first 200 records (20 pages, with 10 results per page) underwent preliminary relevance screening using a predefined stopping rule. This screening step retained 89 potentially relevant records for incorporation into the formal screening process, while 111 records that were clearly outside the predefined scope of the review were excluded at this preliminary stage. This predefined screening limit was adopted to provide a transparent and reproducible stopping rule while maintaining Google Scholar as a supplementary rather than standalone search source [16].

Sources published in English between January 2000 and June 2026 were considered to capture both foundational research and recent advances.

The database searches were conducted between 15 May and 24 June 2026, with 24 June 2026 representing the final search date. The searches yielded 156 records from Web of Science, 112 from Scopus, and 200 screened from Google Scholar, for 468 records overall. Duplicate records were identified across the three search sources and removed before title and abstract screening, resulting in the removal of 70 records. In addition, 111 Google Scholar records were excluded during preliminary relevance screening, leaving 287 unique records for formal title and abstract screening. The 111 Google Scholar exclusions and the 70 duplicate records were mutually exclusive because the out-of-scope Google Scholar records were removed first, after which duplicate removal was applied to the records entering the formal screening pool.

Title and abstract screening and subsequent full-text eligibility assessment were conducted by one reviewer (U.A.) using the predefined eligibility criteria described below. Because screening was performed by a single reviewer, no inter-reviewer disagreement-resolution procedure was required.

For the core evidence synthesis, sources were considered eligible when they addressed the genetic improvement of feed efficiency in aquaculture species, including evidence relating to heritability, genetic correlations, quantitative trait loci (QTL), genome-wide association studies (GWASs), genomic selection, marker-assisted selection, selective breeding, or gene-editing approaches such as CRISPR-Cas9. Authoritative reports and scholarly book chapters were additionally considered when they provided directly relevant contextual, methodological, policy, regulatory, or industry-level information required for the broader narrative synthesis. Sources were excluded from the core evidence synthesis when they focused solely on nutrition, feed formulation, or management interventions without a relevant genetic component, were unrelated to aquaculture species, were not published in English, or could not be retrieved in full text. Therefore, restricting the search to English-language sources may have introduced language bias.

Of the 287 unique records subjected to title and abstract screening, 198 were excluded because they did not meet the predefined eligibility criteria, leaving 89 records for full-text retrieval and assessment. All 89 full-text reports were successfully retrieved. Following full-text assessment, four reports were excluded: two because they did not provide genetic data relevant to feed efficiency and two because they focused exclusively on nutritional or management interventions without a relevant genetic component. No reports were excluded at this stage for the absence of an aquaculture species. Consequently, 85 sources satisfied the eligibility criteria through the database and supplementary Google Scholar search pathway.

To identify relevant publications that might not have been captured by the electronic searches, the reference lists of the 85 eligible sources were subsequently examined through backward citation searching. Candidate sources identified through this supplementary procedure were assessed by U.A. using the same eligibility framework applied to the main search, including relevance to the genetic improvement of feed efficiency in aquaculture or to the predefined contextual scope of the narrative synthesis. Duplicate and clearly irrelevant records were not included. This supplementary procedure resulted in the inclusion of 26 additional eligible sources. Because records retrieved from Web of Science, Scopus, and Google Scholar were merged and deduplicated before formal screening, source-level attribution to an individual electronic database was not retained after deduplication. For this reason, the review reports aggregate counts for the electronic-search pathway and backward citation-searching pathway rather than assigning each included source to a single electronic database.

The 85 sources retained through the electronic-search pathway and the 26 sources identified through backward citation searching were evaluated using the same eligibility criteria, giving a total of 111 eligible sources after the literature-selection process. These sources comprised peer-reviewed journal articles and, where relevant, authoritative reports and scholarly book chapters.

**Figure 1:**
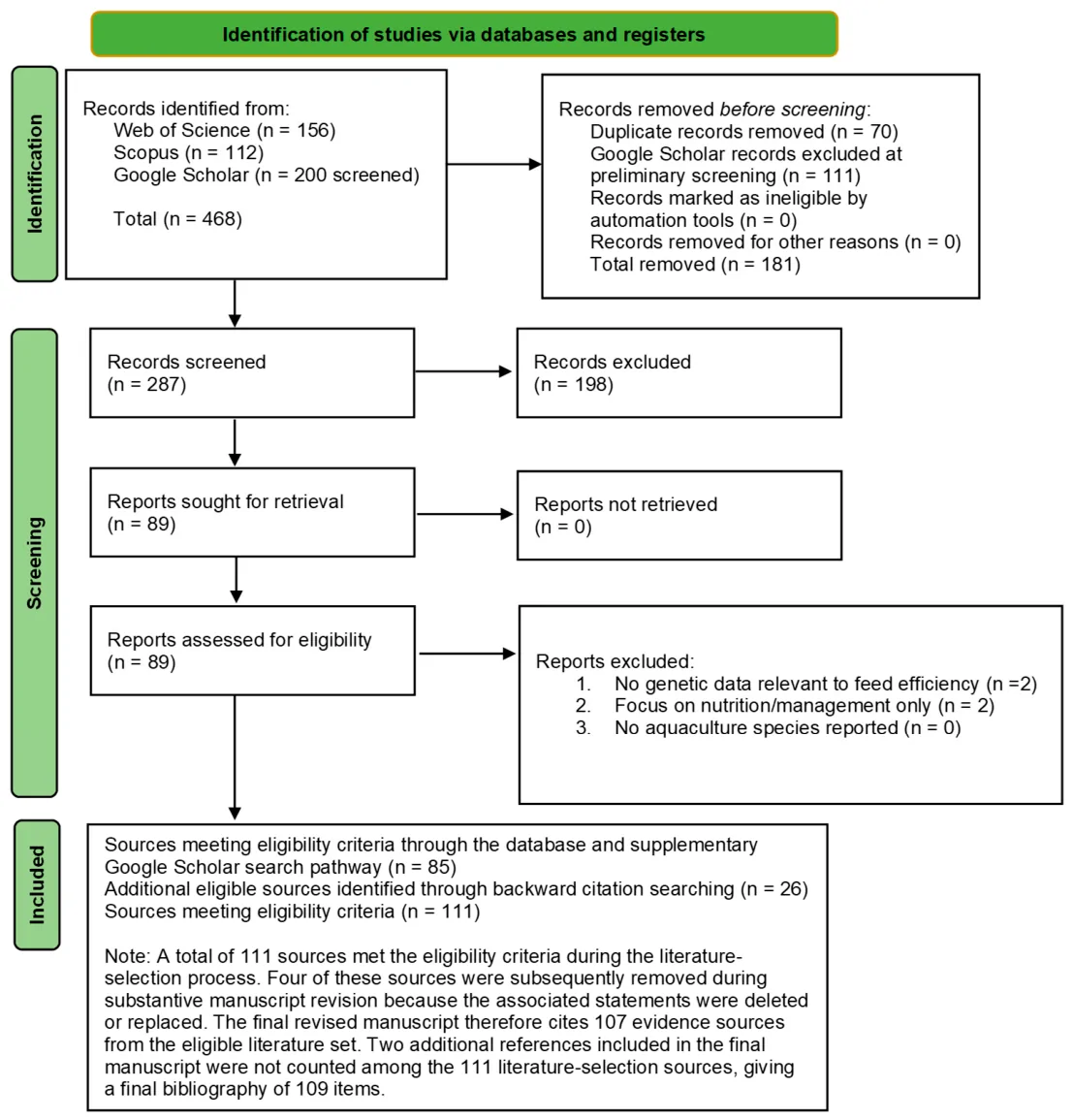
PRISMA-style flow diagram of the literature identification, screening, eligibility assessment, and source-selection process. A total of 468 records were identified from Web of Science (n = 156), Scopus (n = 112), and Google Scholar (n = 200 screened; n = 89 retained after preliminary screening). Duplicate records (n = 70) and Google Scholar records excluded at preliminary screening (n = 111) were removed before formal screening, leaving 287 unique records. After title/abstract screening (n = 198 excluded) and full-text assessment (n = 4 excluded), 85 sources met the eligibility criteria through the database and supplementary Google Scholar search pathway. Backward citation searching identified 26 additional eligible sources, giving a total of 111 sources meeting the eligibility criteria. During substantive manuscript revision, four of these sources were subsequently removed because the associated statements were deleted or replaced with more directly relevant evidence. The final revised manuscript therefore cites 107 evidence sources from the eligible literature set plus two additional references that were not counted among the literature-selection sources, giving a final bibliography of 109 items. The PRISMA-style diagram was used solely to enhance transparency and reproducibility and does not imply adherence to full systematic-review methodology.

During substantive manuscript revision, four of the 111 eligible sources were subsequently removed because the associated statements were deleted or replaced with more directly relevant evidence. The final revised narrative therefore cites 107 evidence sources from the eligible literature set. In addition, two references included in the final manuscript were not counted among the 111 sources identified through the literature-selection process, including Haddaway et al. [16], which was used to justify the Google Scholar search approach, and one additional source introduced during manuscript revision. The final reference list therefore contains 109 items: 107 evidence sources and two additional references that were not counted among the literature-selection sources. The literature identification, screening, eligibility assessment, and source-selection process is summarized in the PRISMA-style flow diagram presented in Figure 1.

## 3. Genetic Framework of Feed Efficiency

The genetic framework of feed efficiency refers to the genetic factors underlying variation in FE, including heritability, genetic correlations with other traits, and interactions with environmental factors [12]. A thorough understanding of the genetic framework is necessary to design effective breeding strategies [10].

Table 1 presents selected illustrative examples of reported heritability estimates for feed-efficiency traits in aquaculture and is not intended to provide an exhaustive summary across species. The reported estimates range from low to high and should be compared cautiously because they differ in trait definition, sample size, population structure, phenotyping method, rearing conditions, and statistical model. In juvenile Nile tilapia, the heritability of the feed conversion ratio (FCR) was estimated at 0.32 ± 0.11 using video-based assessment of individual feed intake in group-reared fish [13]. In Atlantic salmon smolts, FCR heritability varied across three measurement periods in fish evaluated using X-ray-based individual feed-intake phenotyping [14]. In Pacific white shrimp, residual feed intake (RFI) showed a heritability estimate of 0.743 ± 0.157 under a fishmeal-free plant-based diet, although this estimate should be interpreted cautiously because common environmental effects could not be estimated in the study [15]. In juvenile turbot, genotypic RFI heritability was estimated at 0.43 using an individual-cage phenotyping system, although a standard error was not reported for this estimate [12]. Moderate-to-high heritability of residual feed intake has also been reported in Pacific abalone (*Haliotis discus hannai*), with estimates of 0.32 and 0.40 across two measurement periods, further supporting the potential for genetic improvement of feed efficiency across diverse aquaculture taxa [17]. Comparisons among these estimates should be interpreted cautiously because differences in species, traits, diets, rearing systems, and phenotyping approaches can affect the magnitude and precision of genetic parameter estimates [7]. Nevertheless, the available evidence demonstrates measurable genetic variation in feed-efficiency traits across aquaculture species, supporting their potential for genetic improvement while emphasizing the importance of appropriate, species-specific phenotyping.

The direction and magnitude of correlated responses to selection for FE depend on the genetic relationships between feed-efficiency traits and other economically important traits [18]. A strong negative genetic correlation coefficient of (−0.69 ± 0.16) exists between FCR and daily weight gain in juvenile Nile tilapia, indicating that faster growth was associated with improved (lower) FCR [19]. Growth and feed-efficiency traits can be genetically correlated in aquaculture species, although the magnitude and direction of these relationships depend on the FE metric, species, and population [20,21]. The importance of direct selection for FE is supported by the fact that the strength of this relationship varies and that selection for growth alone does not always result in optimal FE improvements [22]. Additionally, genetic correlations with fillet yield are positive; higher fillet yield is linked to improved FE, most likely because more efficient individuals have less visceral fat deposition [23].

Four fundamental physiological processes, which include nutrient digestion and absorption, protein turnover, feed intake regulation, and metabolic partitioning of nutrients, contribute to biological variation in FE [24]. While genetic variations in intestinal enzyme activity and intestinal structure impact absorption efficiency, variation in appetite regulation mediated by neuropeptide signaling pathways contributes to variability in feed intake [25]. Multi-omics evidence from spotted knifejaw has linked variation in feed-conversion efficiency with pathways involved in energy metabolism, nutrient utilization, and related metabolic processes [26].

Genotype-by-environment (G × E) interactions are an important consideration in aquaculture breeding because environmental conditions can alter genetic parameters and the relative performance of families for economically important traits [27]. Significant G × E interactions for FE across different production conditions or feed formulations have been reported in numerous studies. For instance, the genetic ranking of families for FE varies between diets based on fishmeal and those based on plant ingredients, suggesting that specific breeding plans are needed to improve FE across different feed compositions [15].

In crustaceans, G × E interactions have been most extensively studied in Pacific white shrimp (*Litopenaeus vannamei*) [28]. Genetic correlations for residual feed intake (RFI) between plant-based and fishmeal-based diets were estimated at 0.646 ± 0.162, indicating a moderate genotype-by-diet interaction [15]. Additionally, the phenotypic independence of RFI from the growth and maintenance variables included in its regression model is a mathematical property of its estimation; however, this does not necessarily imply genetic independence. Consistent with this distinction, weak to negligible genetic correlations between RFI and average daily gain (0.135 ± 0.204 and −0.018 ± 0.128 across the two growth periods) were reported in *Litopenaeus vannamei* [29], indicating that selection for reduced RFI may have limited effects on growth performance. In turbot (*Scophthalmus maximus*), a genetic correlation point estimate of (*r_g_* = 0.00) between RFI and body weight was reported [12]. Because the study did not report a standard error or confidence interval for this estimate, the value should be interpreted cautiously and should not, on its own, be taken as definitive evidence of complete genetic independence. Nevertheless, the result is consistent with the theoretical objective of RFI as a trait that accounts for differences in maintenance and growth requirements and may therefore provide a useful selection criterion for feed efficiency in turbot. Further studies with independent populations and appropriate measures of uncertainty are needed to confirm the stability of this relationship. In Nile tilapia, a negative genetic correlation between RFI and body-weight gain (*r_g_* = −0.63 ± 0.17) was estimated using a genomic relationship matrix, indicating that selection for reduced RFI may also be associated with a favorable correlated response in growth within the evaluated population [30]. This finding suggests that the genetic independence of RFI from growth may not be universal across species and populations. Thus, while RFI is a preferred parameter for genetic investigations of feed efficiency, its genetic relationship with growth should be verified on a species-specific basis before being assumed. Furthermore, the genetic correlation for feed efficiency ratio (FER) between individual rearing used for phenotyping and group rearing representative of commercial production was estimated at 0.786 [28]. This relatively high correlation indicates some genotype-by-environment interaction but also suggests that genetic rankings for FER were broadly consistent across the two rearing conditions. These findings reinforce the importance of accounting for both dietary and physical production environments in FE genetic improvement programs across species. Additionally, genotype-by-environment interactions have been reported across temperature and management environments in aquaculture species, emphasizing the importance of evaluating genetic performance under production-relevant conditions [31,32,33,34].

The thermal environment can influence genetic parameters for feed-efficiency traits and alter the relative performance of genotypes across temperatures [31]. In the large yellow croaker (*Larimichthys crocea*), feed-efficiency phenotypes were evaluated in the same group of fish at 15 °C, 19 °C, and 24 °C, revealing temperature-specific SNPs and candidate genes associated with FCR and RFI [32]. At 15 °C, 6 SNPs were associated with FCR and 3 with RFI; at 19 °C, no SNPs were associated with FCR, whereas 7 were associated with RFI; and at 24 °C, 7 SNPs were associated with FCR and 6 with RFI [32]. This temperature-dependent genetic architecture suggests that selection for FE at one temperature may not translate to optimal performance at another.

Management and rearing conditions can modify genotype-by-environment interactions for economically important traits in Pacific white shrimp. Improvements in stocking density, aeration, water quality, feed, and feeding regimes have been proposed as approaches to reduce environmental heterogeneity and associated G × E effects [33]. Genetic correlations for growth traits between pond and tank environments were moderate (0.60–0.65), while heritability estimates for body weight differed between environments (h^2^ = 0.68 in ponds and 0.37 in tanks) [33].

Production conditions can influence genetic parameters and genotype-by-environment interactions for growth and feed-efficiency-related traits. In Chinook salmon (*Oncorhynchus tshawytscha*), these interactions were evaluated under low- and moderate-flow regimes [34]. Additionally, in salmonids, G × E interactions can occur across different rearing conditions, including changes in salinity, temperature, and flow regimes [34]. Consequently, environments with phenotype feed-efficiency traits should, as far as practically feasible, reflect the conditions encountered in commercial production. This is particularly important when G × E interactions alter genetic rankings, because selection under substantially different experimental conditions may not accurately predict relative performance in commercial systems [34].

## 4. Molecular Dissection

The molecular dissection of FE traits aims to identify biological pathways, genomic regions, and candidate variants underlying variation in feed efficiency, thereby providing a basis for functional validation and the development of genomic or marker-assisted breeding strategies [35,36].

Genomic regions associated with feed-efficiency-related traits have been identified in several aquaculture species through QTL mapping and genome-wide association approaches [35,36]. Genome-wide association approaches have also been applied to dissect economically important growth-related traits in aquaculture species, further demonstrating the utility of GWASs for identifying candidate genomic regions and genes relevant to breeding [37]. A transcriptomic comparison of high- and low-RFI Jian carp identified 830 differentially expressed genes, with notable enrichment of pathways related to autophagy and fatty acid metabolism [38]. The enrichment of fatty acid metabolism pathways in high- and low-RFI Jian carp suggests that lipid metabolic processes may contribute to variation in feed efficiency [38]. Similarly, in *Litopenaeus vannamei* fed a plant-based diet, transcriptomic and weighted gene co-expression network analysis identified tissue-specific gene modules associated with RFI, with seven hub genes subsequently validated as differentially expressed between high- and low-feed-efficiency groups [39]. Recent genomic evidence in Nile tilapia indicates that feed-efficiency-related traits have a predominantly polygenic architecture, supporting the use of genomic selection models that capture genome-wide marker effects rather than relying on individual QTLs [30]. GWASs can provide relatively strong evidence for genomic regions associated with feed-efficiency traits when studies use adequate sample sizes, dense genome-wide marker coverage, and appropriate statistical thresholds [36,40]. In Atlantic salmon, a GWAS of 700 individuals genotyped using a 55K SNP array identified nine chromosome-wide significant and independent QTLs for feed intake and feed-efficiency traits. Four QTLs were associated with individual feed intake, while five were associated with feed-efficiency traits, including feed conversion ratio and residual feed intake. No large-effect QTLs were detected, supporting a predominantly polygenic architecture for these traits [40]. Similarly, in Nile tilapia, a GWAS using a 65K SNP array revealed a polygenic architecture for feed-efficiency traits, with significant QTLs identified for body-weight gain and feed intake on chromosomes 5 and 7, respectively [30]. In large yellow croaker (*Larimichthys crocea*), a GWAS identified 11 significant SNPs distributed across nine chromosomes that were associated with feed-efficiency traits under a fishmeal-free diet [41]. These GWAS findings are considered robust due to their reliance on genome-wide marker coverage and statistical rigor, although functional validation of the implicated genes remains necessary [30,36,40,41].

Candidate-gene studies have provided preliminary evidence linking genes involved in growth, appetite regulation, and energy metabolism with production and feed-utilization traits in aquaculture, although the strength of evidence varies among genes and species. In Jian carp (*Cyprinus carpio* var. Jian), polymorphisms in the leptin genes *jlLEP-A1* and *jlLEP-A2* were significantly associated with several growth traits, supporting their potential relevance to genetic improvement, although the study did not directly evaluate feed-efficiency traits [42]. In common carp (*Cyprinus carpio*), polymorphisms in the growth hormone receptor (*ghr*) gene have also been investigated in relation to feed conversion and feed-conversion efficiency; however, the reported differences among genotypes were not statistically significant, indicating that the available evidence does not establish a significant association between *ghr* polymorphisms and feed efficiency in this population [43]. These findings illustrate the potential relevance of candidate genes involved in endocrine and metabolic regulation while also emphasizing the need for larger populations and functional validation before such associations can be considered reliable targets for feed-efficiency improvement.

Consistent tissue-specific expression differences associated with divergent feed-efficiency phenotypes have been reported in aquaculture species. In gilthead sea bream (*Sparus aurata*), transcriptomic profiling of fish with divergent individual feed conversion ratios identified differential expression of genes involved in lipid metabolism, mitochondrial activity, oxidative stress, energy sensing, and muscle growth, providing evidence for tissue-specific molecular mechanisms underlying variation in feed efficiency [44]. In contrast, much of the current evidence concerning non-coding RNAs and feed efficiency comes from livestock rather than aquaculture species. Reviews of livestock research have implicated microRNAs, long non-coding RNAs, and circular RNAs in the regulation of energy metabolism processes, including glucose metabolism, adipogenesis, and mitochondrial activity [45]. These findings provide useful mechanistic hypotheses for aquaculture but should not be interpreted as direct evidence of feed-efficiency regulation in fish. Moreover, most reported that non-coding RNA associations remain correlative, and their causal roles in regulating feed efficiency in aquaculture species require further functional validation.

Molecular dissection of FE still has significant limitations despite substantial progress [46]. Because feed-efficiency traits are generally polygenic and mapping resolution remains limited in many aquaculture populations, QTLs often span broad genomic regions, and relatively few causal variants have been functionally confirmed [36,47]. To confirm the causal role of discovered variants and genes in aquatic animals, functional validation is necessary; novel gene-editing methods are beginning to address this gap [48]. Overall, the molecular dissection of feed efficiency in aquaculture has yielded a rich but heterogeneous body of evidence. GWAS-derived QTLs from well-powered studies represent the most robust findings [36]. Candidate-gene associations, transcriptomic markers, and non-coding RNA signatures provide valuable hypotheses regarding the biological mechanisms underlying feed efficiency; however, much of this evidence remains associative or correlative, and causal relationships require further functional validation in aquaculture species.

## 5. Selective Breeding Approaches

Traditional selective breeding is an established approach for improving FE in aquaculture and has demonstrated long-term genetic benefits within breeding programs [22]. Selective breeding and structured crossing strategies continue to be applied in aquaculture to exploit genetic variation in economically important production traits [49].

Since it is difficult to measure individual feed intake on a large number of selection candidates, family-level averages are employed to estimate breeding values in traditional selective breeding for FE [22]. To limit inbreeding and maintain family representation, aquaculture breeding programs commonly use family-based selection strategies, with breeding values estimated from phenotypic and pedigree or genomic information collected from relatives and selection candidates [47]. Genetic relationships between growth and feed efficiency have been reported in both livestock and aquaculture species, although their magnitude varies among populations and traits. In beef cattle, negative genetic relationships between growth-related traits and FCR have been reported, indicating that faster growth can be associated with improved feed efficiency [50]. Similar patterns have been observed in aquaculture species, including Nile tilapia (*r_g_* = −0.69 ± 0.16) [19]. However, the strength of this relationship varies across species and production systems.

Indirect selection for FE through growth rate can be considered when the two traits are genetically correlated [51]. When reliable individual feed-intake phenotypes are available, direct selection on feed-efficiency traits can provide a more targeted response than indirect selection through correlated traits such as growth [22]. Indicator traits such as residual body-weight gain and carcass or visceral traits may provide useful proxies for FE when direct measurement of individual feed intake is not practical [8]. In rainbow trout, indirect selection against lipid deposition has also been shown to improve feed conversion ratio, illustrating the potential value of correlated indicator traits when direct measurement of feed intake is difficult [52].

To balance FE improvement with other breeding goals, including growth, disease resistance, fillet quality, and reproductive performance, breeding objectives can incorporate information from multiple economically important traits [53,54]. The relative weighting assigned to feed-efficiency-related traits depends on the breeding objective, available phenotypes, genetic parameters, and production economics and therefore should not be generalized across breeding programs. In Atlantic salmon, weight gain and feed intake have been evaluated as economically important breeding-goal traits, and selection-index strategies incorporating feed-efficiency-related information have been assessed for improving feed efficiency [23].

Selective breeding programs in Nile tilapia have produced substantial improvements in growth, feed conversion, and survival across improved strains [55]. For the genetically improved Abbassa Nile tilapia (GIANT) strain, on-station comparisons reported approximately 28% faster growth than a commercial strain, while a subsequent farm-level impact assessment found a 15.7% lower FCR relative to commercial strains [55,56]. While FCR is not always used as a direct selection criterion because individual feed intake is difficult to measure at scale, feed-efficiency-related traits can be incorporated into multi-trait breeding objectives when sufficiently reliable phenotypic and genetic information is available [23]. Long-term selective breeding has produced substantial improvements in feed efficiency and related production traits in aquaculture species, including Nile tilapia and rainbow trout [56,57]. In rainbow trout, ten generations of multi-trait selection produced substantial realized gains, including a 17–20% reduction in feed conversion ratio, providing direct evidence that sustained selective breeding can generate cumulative improvements in feed efficiency [58]. Together, these findings demonstrate that long-term selective breeding can produce substantial cumulative genetic gains in feed efficiency and related production traits in aquaculture [22,56,57].

## 6. Genomic Selection and Marker-Assisted Strategies

Compared with conventional pedigree-based selection, genomic selection (GS) can improve the accuracy of breeding-value prediction for complex traits in aquaculture [59,60]. Although GS can reduce the need to phenotype every selection candidate, it does not eliminate the requirement for phenotyping. Genomic prediction relies on a suitably sized reference or training population in which individuals are both phenotyped for the target traits and genotyped using genome-wide markers [36,59]. Prediction equations developed from this reference population can then estimate genomic estimated breeding values (GEBVs) for genotyped selection candidates that may not have direct phenotypic records. To maintain prediction accuracy across generations and production environments, continued phenotyping, periodic updates to the reference population, and appropriate cross-validation or independent validation are necessary. The size and composition of the training population, marker density, phenotypic quality, and statistical prediction model are therefore critical design elements for genomic selection of feed-efficiency traits [59,61].

The advantage of genomic prediction over pedigree-based prediction is trait- and validation-design-dependent. In gilthead sea bream (*Sparus aurata*), individual feed efficiency has also been shown to be heritable and highly polygenic, supporting the potential use of genomic selection for improving feed-efficiency traits in this species [8]. In Nile tilapia, pedigree-based BLUP (PBLUP), genomic BLUP (GBLUP), and single-step GBLUP (ssGBLUP) were directly compared for feed conversion ratio, residual feed intake, feed intake, and body-weight gain using fivefold cross-validation [30]. Genomic models improved prediction accuracy for several feed-efficiency-related traits compared with PBLUP, with improvements of up to 34% reported depending on the trait and validation scheme [30]. A variety of statistical models, including GBLUP and Bayesian approaches such as BayesA and BayesB, have been evaluated for genomic prediction in aquaculture [62]. The relative performance of GBLUP and Bayesian genomic prediction methods depends on factors such as trait architecture, heritability, marker density, and reference-population size, and no single method is consistently optimal across aquaculture traits [36,62,63].

Marker-assisted selection (MAS) can be useful when validated QTLs of sufficiently large and reproducible effect are available for a target trait [35,64]. In common carp, fine-scale QTL mapping identified multiple loci associated with feed conversion efficiency, providing candidate regions that may support future marker-assisted breeding after appropriate validation [64]. However, because feed-efficiency traits are generally polygenic and much of their genetic variation is distributed across numerous loci of small effect, genomic selection is likely to be more broadly applicable than MAS for routine improvement of FE in aquaculture [36]. Thus, MAS is most appropriate for traits or populations in which one or a few validated loci explain a meaningful proportion of genetic variation, whereas genomic selection is better suited to highly polygenic traits for which genome-wide marker effects must be captured simultaneously.

Implementation cost is an important consideration when applying genomic selection in commercial aquaculture breeding programs [62]. Common genotyping approaches include single-nucleotide polymorphism (SNP) arrays and genotyping-by-sequencing (GBS), while genotype imputation can increase effective marker density and reduce genotyping costs [65].

In European sea bass (*Dicentrarchus labrax*), incorporating genomic information improved the reliability of estimated breeding values for feed-efficiency-related traits compared with pedigree-based prediction, with increases in reliability ranging from approximately 10% to 125% depending on the trait and prediction model [66]. More recent response-to-selection evidence in European sea bass has further demonstrated the practical application of genomic selection for individual feed efficiency and associated commercial traits [67]. This finding further illustrates the importance of evaluating genomic prediction under appropriate validation designs. Validation under production-relevant conditions is ultimately necessary to determine whether prediction accuracies observed in experimental populations translate into reliable genetic gains in commercial breeding programs.

## 7. Emerging Biotechnological Interventions

Gene editing, epigenetic approaches, and microbiome-informed strategies represent emerging biotechnological research areas with potential applications for understanding and improving feed efficiency in aquaculture [68,69,70].

Targeted manipulation of genes involved in growth, energy homeostasis, and muscle development through CRISPR-Cas9 provides an opportunity to functionally validate candidate genes and potentially improve feed-utilization traits in aquaculture [71]. In channel catfish (*Ictalurus punctatus*), CRISPR/Cas9-mediated editing of the melanocortin-4 receptor (*mc4r*) was associated with an observed improvement in the feed conversion ratio, with a mean FCR of 1.18 in edited fish compared with 1.57 in control fish; however, this difference was not statistically significant because the FCR assessment was based on only two replicates per group [72]. Similarly, CRISPR/Cas9-mediated knockout of myostatin (*mstn*) in red sea bream (*Pagrus major*) resulted in higher feed efficiency than in wild-type fish, with subsequent evaluation at the early stage showing an approximately 9% improvement in feed efficiency [73,74]. In zebrafish (*Danio rerio*), *pomc* knockout also increased growth and reduced FCR relative to wild-type and heterozygous fish [75]. Together, these studies provide proof-of-concept evidence that targeted genetic modification can influence feed-utilization traits, including FCR and feed efficiency, although the available evidence remains species-specific and further functional validation under production-relevant conditions is required.

Epigenetic regulation is an increasingly studied area in aquaculture and may contribute to variations in metabolic and feed-utilization traits [69]. Environmental and nutritional conditions can alter epigenetic marks such as DNA methylation and histone modifications, although evidence for stable transgenerational inheritance of feed-efficiency-related epigenetic states in aquaculture remains limited [69]. For example, diet-associated differences in genome-wide DNA methylation have been reported in grass carp, with differentially methylated regions linked to pathways involved in muscle growth and metabolism, including PI3K/Akt and Notch signaling [76]. Epigenetic information may eventually complement DNA-sequence-based selection, but the stability, heritability, and predictive value of epigenetic markers across generations and production environments require further validation before routine use in aquaculture breeding programs [69,77].

Variation in feed efficiency has been associated with differences in gut microbiota composition, and heritable variation in gut microbial communities has been reported in aquatic species [70]. Improved FE has been associated with certain gut bacterial taxa that may enhance nutrient absorption and digestion [78]. These findings suggest that host genetic influences on the gut microbiota could eventually provide complementary information for feed-efficiency breeding; however, microbiome-informed selection in aquaculture remains experimental, and its stability, heritability, and predictive utility across populations and production environments require further validation before commercial application [70,79]. These findings suggest that host genetic influences on the gut microbiota could eventually provide complementary information for feed-efficiency breeding; however, microbiome-informed selection in aquaculture remains experimental, and its stability, heritability, and predictive utility across populations and production environments require further validation before commercial application [70,79].

Notwithstanding these advantages, gene-edited aquaculture strains still face significant regulatory and public acceptance issues [80]. Regulatory frameworks for gene-edited organisms vary among countries [81]. Commercial deployment of gene-edited aquaculture breeds with improved FE will depend in part on regulatory approval and public acceptance, both of which remain important considerations for wider adoption [82].

Regulatory approaches to genome-edited animals vary considerably among jurisdictions. Countries such as Argentina, Brazil, and Japan have adopted regulatory pathways under which certain genome-edited animals, particularly those carrying targeted mutations without introduced foreign DNA, may be assessed differently from conventional transgenic organisms [83,84]. These approaches have enabled the commercialization of genome-edited fish in Japan and non-GMO determinations for genome-edited fish in Argentina and Brazil [83,84]. In contrast, the European Union generally applies a more restrictive regulatory approach, with many genome-editing applications remaining within the regulatory framework governing genetically modified organisms [83]. In the United States, intentional genomic alterations in animals are subject to regulatory oversight, with the level of regulatory scrutiny depending on the characteristics, intended use, and potential risks associated with the genomic alteration [83,84]. China continues to regulate genome-editing applications within its broader agricultural biotechnology framework, although regulatory pathways remain under development [83]. Collectively, these differences demonstrate that the commercial application of genome-edited aquaculture species depends not only on the biological characteristics of the edit but also on the regulatory framework of the jurisdiction in which commercialization is sought.

Consumer acceptance remains an important consideration for the commercialization of genome-edited aquaculture products, and attitudes may vary according to consumers’ knowledge of genome-editing technology, perceived benefits and risks, and the information provided about the resulting products. Evidence from Japan indicates that providing understandable information about the purpose, potential benefits, and safety assessment of genome-edited foods can increase consumer acceptance [85,86]. Nevertheless, acceptance should not be assumed to be uniform across products or populations, and concerns regarding environmental risks, food safety, and the nature of the genetic alteration may influence consumer responses [84]. Therefore, transparent communication, appropriate risk assessment, and effective risk-mitigation strategies are important considerations for the responsible introduction of genome-edited aquaculture products.

The escape of gene-edited fish from aquaculture facilities into natural ecosystems represents a primary ecological concern [82]. The main risks include gene flow resulting in the introgression of edited alleles into wild populations, with potentially harmful genetic and ecological consequences [82]. Should gene-edited fish escape, there is potential for ecological and genetic impacts on wild populations, including competition for resources, altered predation dynamics, and disruption of native genetic diversity [82].

Gene editing has the potential to improve animal welfare in aquaculture, particularly through applications such as enhanced disease resistance [48]. However, empirical welfare data for gene-edited fish remain limited, and potential welfare effects should be evaluated on a case-by-case basis [48,82]. In addition, the stability of edited traits across generations requires further assessment before widespread commercial application [82]. Off-target effects, mosaicism, and unintended impacts on other important traits are important technical risks that must be carefully evaluated [48,87].

## 8. Implementation Challenges and Knowledge Gaps

The rate of genetic improvement for FE in aquaculture remains constrained by substantial challenges and knowledge gaps, despite considerable progress [35,88].

The number of aquatic animals that can be phenotyped for individual feed intake is limited by the labor-intensive and costly nature of conventional phenotyping approaches, including imaging- and X-radiography-based methods [89,90]. Limited phenotyping capacity can reduce the statistical power of genetic-association studies and constrain the size of reference populations available for genomic prediction [36,59,62]. Emerging high-throughput phenotyping approaches, including near-infrared spectroscopy and X-radiography-based methods, have the potential to increase the efficiency and scale of feed-intake phenotyping in aquaculture [89,90].

Unequal availability and quality of genomic resources across aquaculture species remain important constraints on the wider implementation of genomic approaches in breeding programs. High-quality reference genome assemblies and genome-wide marker resources are available for several major aquaculture species, including Atlantic salmon (*Salmo salar*), Nile tilapia (*Oreochromis niloticus*), and common carp (*Cyprinus carpio*), whereas genomic resources remain less developed or less extensively applied in many other cultured species [36]. Moreover, the availability of a reference genome alone does not ensure comprehensive functional annotation, and further characterization of regulatory and functional genomic elements is required to improve the biological interpretation and application of genomic information in aquaculture breeding [91].

Another significant challenge is integrating genetic improvement for FE with the transition toward alternative feed sources. Plant-based diets and other alternative protein sources, including insect meal and microbial protein, are increasingly being evaluated as replacements for fishmeal-based diets in aquaculture [92]. Genetic responses to feed-efficiency-related traits can depend on diet composition; for example, in Pacific white shrimp, genetic correlations between plant-based and fishmeal-based diets were estimated at 0.646 ± 0.162 for residual feed intake and 0.549 ± 0.163 for daily feed intake, indicating genotype-by-diet interactions [15]. In gilthead sea bream, selected high-growth fish also showed improved utilization of diets containing insect meal, single-cell protein, and microalgal oil, illustrating that genetic background can influence performance under novel feed formulations [93]. These findings support evaluating feed-efficiency traits under the target production diet rather than assuming that genetic performance will remain unchanged across feed formulations.

Lastly, balancing genetic gain with the maintenance of genetic diversity and control of inbreeding remains an important long-term challenge in aquaculture breeding programs [35,53]. Strong selection for FE and other traits may result in increased inbreeding and reduced genetic diversity, thereby constraining long-term genetic gain and increasing the risk of inbreeding depression in fitness-related traits [35,94]. Optimal strategies for balancing these objectives are still being evaluated in aquaculture breeding programs, although genomic selection offers new tools for controlling inbreeding while maintaining high rates of genetic gain [62]. Genotype-by-environment interactions can reduce the consistency of genetic performance across rearing conditions, making it important to evaluate selection candidates under production-relevant environments [31,34].

## 9. Future Perspectives

Genetic enhancement of FE is an important component of sustainable aquaculture because improvements in feed utilization can provide both economic and environmental benefits [22,95]. Knowledge of the genetic architecture of economically important traits, the development of genomic selection approaches, and the application of gene-editing tools have advanced substantially in aquaculture [36,48]. The integration of emerging technologies is expected to create new opportunities for the aquaculture industry and may accelerate genetic gain for FE [35,95].

While an integrated breeding pipeline represents a potential future direction for feed-efficiency improvement, the availability and application of genomic resources vary substantially among major aquaculture species, and these differences are better described by the specific tools and evidence available than by broad categories of genomic “readiness.” Atlantic salmon has established high-density SNP genotyping platforms, and recent genomic studies have directly investigated feed intake and feed-efficiency traits [40,96]. Nile tilapia likewise has well-established SNP-based genomic tools and direct evidence that genomic prediction can improve the evaluation of feed-efficiency traits compared with pedigree-based prediction [30]. Common carp has high-density SNP resources and mapped quantitative trait loci associated with feed-conversion efficiency, although routine genomic selection specifically targeting feed efficiency is less extensively documented [64]. The large yellow croaker has recently been evaluated using genome-wide association and genomic prediction analyses for feed-efficiency traits under a fishmeal-free diet [41]. Pacific white shrimp has also demonstrated improved genetic evaluation of feed-efficiency traits using genomic information, with genomic prediction outperforming pedigree-based approaches in the evaluated population [97]. Gilthead sea bream has a validated high-density SNP array suitable for genomic applications and selective breeding, although direct genomic-selection studies specifically targeting feed efficiency remain comparatively limited [98]. Rainbow trout possesses well-developed genomic resources, including a validated high-density SNP array, and established genomic-selection applications across economically important traits, although direct FE-specific genomic evidence remains less extensive than the broader genomic-selection literature in salmonids [99,100]. Table 2 presents a concise comparison of the available genomic resources and FE-specific genomic evidence across these major aquaculture species. Accordingly, the applicability of genomic selection for feed efficiency should be evaluated using species-specific evidence on reference genomes, marker platforms, breeding populations, genomic prediction studies, and direct FE-related genomic data.

For species with limited genomic resources, the priority should be to establish basic population-structure information and develop low-density marker panels [10]. Low-density genotyping (1K–5K SNPs) followed by genotype imputation offers a cost-effective entry point [102]. For example, a 1K SNP array for tambaqui (*Colossoma macropomum*) was evaluated for genotype imputation, providing a low-density genomic tool for this species [103]. Similarly, genotype imputation from very low-density panels has been successfully demonstrated across Atlantic salmon, turbot, common carp, and Pacific oyster [104]. These strategies can reduce genotyping costs significantly and facilitate broader uptake of genomic selection.

The advancement of FE breeding for minor and understudied species will be accelerated by international scientific collaboration and open data exchange [35,105].

Automated phenotyping methods could increase phenotyping throughput and support the collection of larger datasets for genomic prediction in aquaculture [106]. Multi-omics information, including transcriptomic, epigenomic, and microbiome data, may provide complementary biological information that could eventually be integrated with genomic data to improve the characterization and prediction of complex traits in aquaculture [107,108]. Larger and well-characterized reference populations, together with standardized and reliable phenotyping procedures, are important for improving the comparability and accuracy of genomic prediction in aquaculture breeding programs [36,62]. Broader realization of the benefits of genetic improvement for FE will require strategic investment in genomic resources, breeding infrastructure, and technical capacity, particularly in regions where limited resources currently constrain the adoption of advanced genetic technologies [105].

CRISPR-Cas9-based functional validation of candidate genes may help establish their causal roles in economically important traits and thereby inform future genetic-improvement strategies for FE in aquaculture [48,109].

In summary, feed efficiency has the potential to provide substantial economic and environmental benefits, making it an important target trait for genetic improvement in aquaculture [22,95]. Approaches to FE improvement have evolved with advances in quantitative genetics, genomics, and biotechnology [35,36]. Traditional indirect selection through growth is increasingly being complemented by direct selection, genomic prediction, and targeted gene-editing approaches [22,36,48]. These approaches have considerable potential to improve the economic efficiency and environmental sustainability of aquaculture when appropriately integrated within breeding programs [35,95].

## 10. Conclusions

Feed efficiency is an economically and environmentally important breeding target in aquaculture because improvements in feed utilization can reduce production costs and nutrient losses while supporting more sustainable production. Available evidence indicates that FE-related traits show exploitable genetic variation in several major aquaculture species, although heritability estimates, genetic correlations, and responses to selection vary according to species, population, diet, phenotyping method, and production environment. Conventional selective breeding remains important, while genomic selection provides additional opportunities to improve prediction accuracy for complex FE traits where suitable phenotypic and genomic resources are available.

Recent advances in GWASs, transcriptomics, high-throughput phenotyping, and genomic prediction have improved our understanding of the biological and genetic basis of FE; however, most candidate genes and molecular associations still require direct functional validation. Gene editing, epigenetic approaches, microbiome-associated approaches, and multi-omics integration therefore remain promising but comparatively less mature components of FE improvement programs. Their practical application will depend on stronger causal evidence, cost-effective phenotyping, appropriate genomic resources, validation under commercial farming conditions, and regulatory acceptance.

Overall, future genetic improvement of feed efficiency is likely to depend on integrating accurate phenotyping, quantitative genetics, genomic prediction, and validated molecular information within species-specific breeding programs. Rather than applying a single technological pathway across all aquaculture species, breeding strategies should reflect the genomic resources, production systems, economic priorities, and genotype-by-environment interactions relevant to each species.

## Figures and Tables

**Table 1:** Selected examples of reported heritability estimates for feed-efficiency traits in aquaculture species.

Species	Sample Size/Conditions	FE Trait	Heritability (h^2^ ± SE)	Study/Model Limitation	Source
Nile tilapia (*Oreochromis niloticus*)	40 families; juvenile group-rearing/video phenotyping	FCR	0.32 ± 0.11	Individual feed intake estimated under group-rearing conditions; estimate specific to the experimental population and phenotyping design	[13]
Atlantic salmon (*Salmo salar*)	700 smolts; X-ray-based individual feed-intake phenotyping at three measurement time points	FCR	0.09 ± 0.04; 0.08 ± 0.04; 0.23 ± 0.06	Heritability varied among measurement periods; estimates depend on the timing and repeated phenotyping design	[14]
Pacific white shrimp (*Litopenaeus vannamei*)	900 individuals; 30 families; fishmeal-free plant-based diet	RFI	0.74 ± 0.16	Common environmental effects could not be estimated; estimate derived under a specific diet	[15]
Turbot (*Scophthalmus maximus*)	2160 juveniles; 72 full-sib families; individual-cage phenotyping	RFI	0.43 (SE not reported)	Standard error/confidence interval not reported; individual-cage conditions may differ from commercial production	[12]

**Table 2:** Genomic resources and FE-specific evidence in selected aquaculture species.

Species	Key Genomic Resource	FE-Specific Genomic Evidence	Representative Source
Atlantic salmon (*Salmo salar*)	High-density SNP arrays and extensive genomic-selection resources	Direct GWAS evidence for feed intake and FE traits	[40,96]
Nile tilapia (*Oreochromis niloticus*)	65K SNP array and structured breeding populations	Direct genomic prediction and GWAS for FCR, RFI and feed intake	[30]
Rainbow trout (*Oncorhynchus mykiss*)	Validated 57K SNP array and established genomic-selection tools	FE-specific genomic evidence remains less extensive than for other production traits	[100,101]
Common carp (*Cyprinus carpio*)	250K SNP array and high-density linkage map	QTL identified for feed-conversion efficiency	[64]
Large yellow croaker (*Larimichthys crocea*)	Genome-wide SNP data and genomic prediction tools	Direct GWAS and genomic prediction for FE under fishmeal-free diets	[41]
Pacific white shrimp (*Litopenaeus vannamei*)	Genome-wide SNP information used in genomic evaluation	Direct genomic prediction for FER and RFI	[97]
Gilthead sea bream (*Sparus aurata*)	Validated 60K MedFish SNP array	Direct FE-specific genomic-selection evidence remains limited	[98]

## Data Availability

No new data were created or analyzed in this study. Data sharing is not applicable to this article.
